# SperoPredictor: An Integrated Machine Learning and Molecular Docking-Based Drug Repurposing Framework With Use Case of COVID-19

**DOI:** 10.3389/fpubh.2022.902123

**Published:** 2022-06-16

**Authors:** Faheem Ahmed, Jae Wook Lee, Anupama Samantasinghar, Young Su Kim, Kyung Hwan Kim, In Suk Kang, Fida Hussain Memon, Jong Hwan Lim, Kyung Hyun Choi

**Affiliations:** ^1^Department of Mechatronics Engineering, Jeju National University, Jeju, South Korea; ^2^BioSpero, Inc., Jeju, South Korea

**Keywords:** drug repurposing, COVID-19, machine learning, databases, data analytics, host proteomes, molecular docking

## Abstract

The global spread of the SARS coronavirus 2 (SARS-CoV-2), its manifestation in human hosts as a contagious disease, and its variants have induced a pandemic resulting in the deaths of over 6,000,000 people. Extensive efforts have been devoted to drug research to cure and refrain the spread of COVID-19, but only one drug has received FDA approval yet. Traditional drug discovery is inefficient, costly, and unable to react to pandemic threats. Drug repurposing represents an effective strategy for drug discovery and reduces the time and cost compared to *de novo* drug discovery. In this study, a generic drug repurposing framework (SperoPredictor) has been developed which systematically integrates the various types of drugs and disease data and takes the advantage of machine learning (Random Forest, Tree Ensemble, and Gradient Boosted Trees) to repurpose potential drug candidates against any disease of interest. Drug and disease data for FDA-approved drugs (*n* = 2,865), containing four drug features and three disease features, were collected from chemical and biological databases and integrated with the form of drug-disease association tables. The resulting dataset was split into 70% for training, 15% for testing, and the remaining 15% for validation. The testing and validation accuracies of the models were 99.3% for Random Forest and 99.03% for Tree Ensemble. In practice, SperoPredictor identified 25 potential drug candidates against 6 human host-target proteomes identified from a systematic review of journals. Literature-based validation indicated 12 of 25 predicted drugs (48%) have been already used for COVID-19 followed by molecular docking and re-docking which indicated 4 of 13 drugs (30%) as potential candidates against COVID-19 to be pre-clinically and clinically validated. Finally, SperoPredictor results illustrated the ability of the platform to be rapidly deployed to repurpose the drugs as a rapid response to emergent situations (like COVID-19 and other pandemics).

## Graphical Abstract

Illustration of SperoPredictor repurposing framework. It starts with data collection followed by machine learning models training, model deployment, literature, and molecular docking-based validation. 
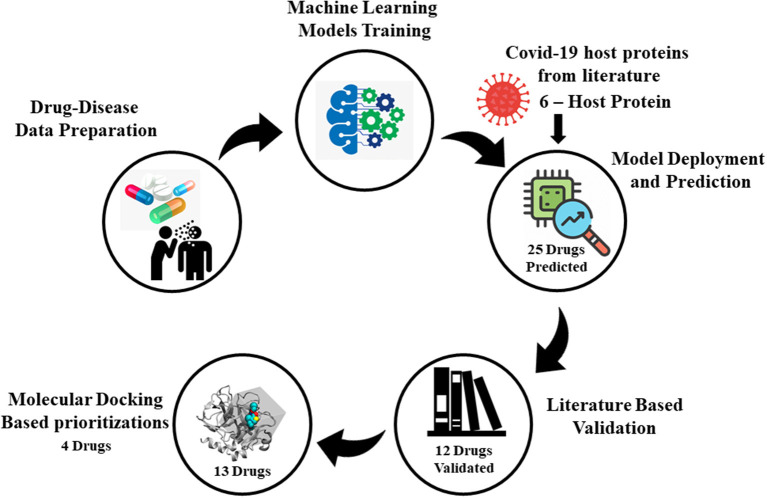

## Introduction

Coronavirus (CoV) normally targets the respiratory tract of humans (mammals), resulting in mild-to-severe respiratory tract infections (1). In the last two decades, severe acute respiratory syndrome (SARS-CoV) and Middle East respiratory syndrome coronavirus (MERS-CoV) referred to as pathogenic human coronaviruses, have caused global epidemics with high mortality and morbidity rates (2) with a burden on the worldwide economy (3). In December 2019, Wuhan (China) experienced the third coronavirus pandemic, called novel coronavirus (SARS-CoV-2 or 2019-nCoV) disease 2019 (4). Over 79,000 confirmed cases with more than 2,600 deaths from COVID-19 or SARS-CoV-2 outbreak worldwide were reported as of 24 February 2020 along with contact transmission due to human-to-human interaction (5). Moreover, according to the World Health Organization (WHO), as of 15 February 2022, there have been a total number of 414.3 M confirmed cases of COVID-19 with 5.846 M deaths worldwide, while the United States, India, and Brazil remain the worst-hit countries (6), with worldwide economic costs of more than $16 trillion. In this connection, studies have been conducted for COVID-19 health and exploratory data analysis for classification, comparative analysis (7), and prediction using machine learning, such as done in (8) specifically targeting the Mexican and Brazilian patients. The results from the study consider data from patients under the age of 0–120 years demonstrating the application of big data technologies along with machine learning. Similarly, the relation between the spread rates of COVID-19 in high-risks countries is also studied in (9) where Pearson correlation was considered to first study the relationship between the countries under study followed by the use of PCA to categorize the countries based on the spread rates of COVID-19. Moreover, a vast number of efforts from various national and international research groups around the world have been made to obtain effective drugs for COVID-19. To date, the FDA has approved only one antiviral (oral) drug for the treatment of COVID-19, and it is regarded as a major step forward in the fight against COVID-19 (10). Owing to the lack of effective oral drugs for COVID-19, there is an urgent need to develop effective treatments for 2019-nCoV or SARS-CoV-2.

Additionally, witnessing the pandemics over the last two decades and the emergence of new diseases, the total money diverted to pharmaceutical and biomedical research has significantly increased, increasing the annual number of novel treatments approved by the US Food and Drug Administration (FDA) slightly (11). As estimated in a study, pharmaceutical companies invested $802 million in 2013 which increased to $2.6 billion in 2015 owing to the development of novel drug molecules approved by the FDA (12). Thus, the development of novel drug molecules until the approval of the FDA requires approximately $2–$3 billion investment and a time of approximately 12–15 years, with < 10% chance of success. This renders drug development a risky process that suffers from high attrition rates, substantial costs, and a slow pace (13). An effective alternative to *de novo* drug development is drug repurposing, the process of determining new indications of the already approved (FDA), failed/abandoned, or investigational drugs for use in other diseases (14). Drug repurposing reduces the time and money spent on *de novo* drug development and clinical trials (15–17) because the safety and efficacy data of the drugs to be repurposed are already known. In addition, sufficient investment of time and money is still required for drug repurposing when opting for experimental approaches (18). The problem has been solved with the advent of computational approaches which have come along with enormous computing power to digest vast amounts of heterogeneous data for the testing of the systematic drug repurposing hypothesis (12, 13, 15–17). Moreover, targeting a single viral protein often results in high drug resistance owing to the rapid evolution of the virus genome (1). Conventional methods based on the three-dimensional structure of proteins and ligands are limited by the unavailability of protein structures that are normally encountered for most viral and human targets. Apart from that disease diagnosis studies are performed, such as done in (19). In the said study, significant features were ranked using a random tree, decision tree, support vector machine (SVM), and chi-squared decision tree models. The study results show the significance of the RT models which ultimately outperform the other models.

Moreover, *in silico* methods that predict drug–target interactions (DTI) or associations, use machine learning to train, test, and validate the machine-learning-based prediction models. DTIs are the pairs of drugs and their targets interacting with each other. Moreover, these trained prediction models are subsequently deployed to discover novel DTIs. The data on which feature-based machine-learning models are trained can be obtained from publicly available online databases or in-house laboratories. The well-known databases for DTIs and target–target interactions are DrugBank (20), KEGG (21), and STITCH (22–24). When discussing the representation of drugs and target data, people have presented them in many different forms; for example, drugs are represented by their chemical structures (25), drug expression profiles (26) side effects (27), and Anatomical Therapeutic Chemical (ATC) codes (28), while targets have been represented using their gene ontology information (28), genome sequence (21), protein–protein interaction (29), and disease associations (30). Moreover, many successful feature-based computational methods have been developed over the past decade. Feature-based methods are standard methods that require feature vectors of a fixed length as input (31). According to the literature, an earlier feature-based method (32) was used for target representation; this method performs binary vector representation of the drugs, indicating the presence or absence of functional groups in the chemical structure of the drugs with amino acid composition. Simply put, if a drug is represented by *d*, then the feature vector of the drug would be [*d*_1_*, d*_2_*, d*_3_*,…, d*_*d*_]. Similarly, for target *t*, it would be [*t*_1_*, t*_2_*, t*_3_*,…, t*_*t*_]. The drug and target feature vectors are subsequently concatenated to form a drug–target pair *(d,t)*. In addition, many feature-based methods have been presented based on random forest (RF) (29, 30), rotation forest (33), and extremely randomized trees (34) that are formed by the ensemble of decision trees. In the given studies, many of the important features are not considered. Specifically, in most studies, only two or three features are focused on, missing the other important information. While in our work, unlike other studies, SperoPredictor® uses multiple drugs (four drugs) and disease (three diseases) features unified in binary strings covering diverse aspects of drugs and diseases data. The use of various features in our study make the classification models more generalized and accurate on unseen data. Additionally, other feature-based methods include relevance vector machines (35), KNN (fuzzy) (36), and deep learning (37–39). Additionally, most of the studies are focusing on single ML model trained and then deployed, while in our study we have trained multiple machine-learning models which are later stacked together to synergize the prediction confidence. Once the predictions are taken from the deployed machine-learning models, the need for further validation of the drugs is highly needed which most of the studies are lacking. Here, a bifold molecular docking validation is performed for the predicted drugs with host COVID-19 proteins. Moreover, there is dire need of well-trained models that are fast, accurate, and ready to be deployed as rapid response, such as SperoPredictor. Further it can also be deployed to predict the drugs for a given disease as input to the deployed model and can find the new indication for drugs given as input.

**Figure 1:**
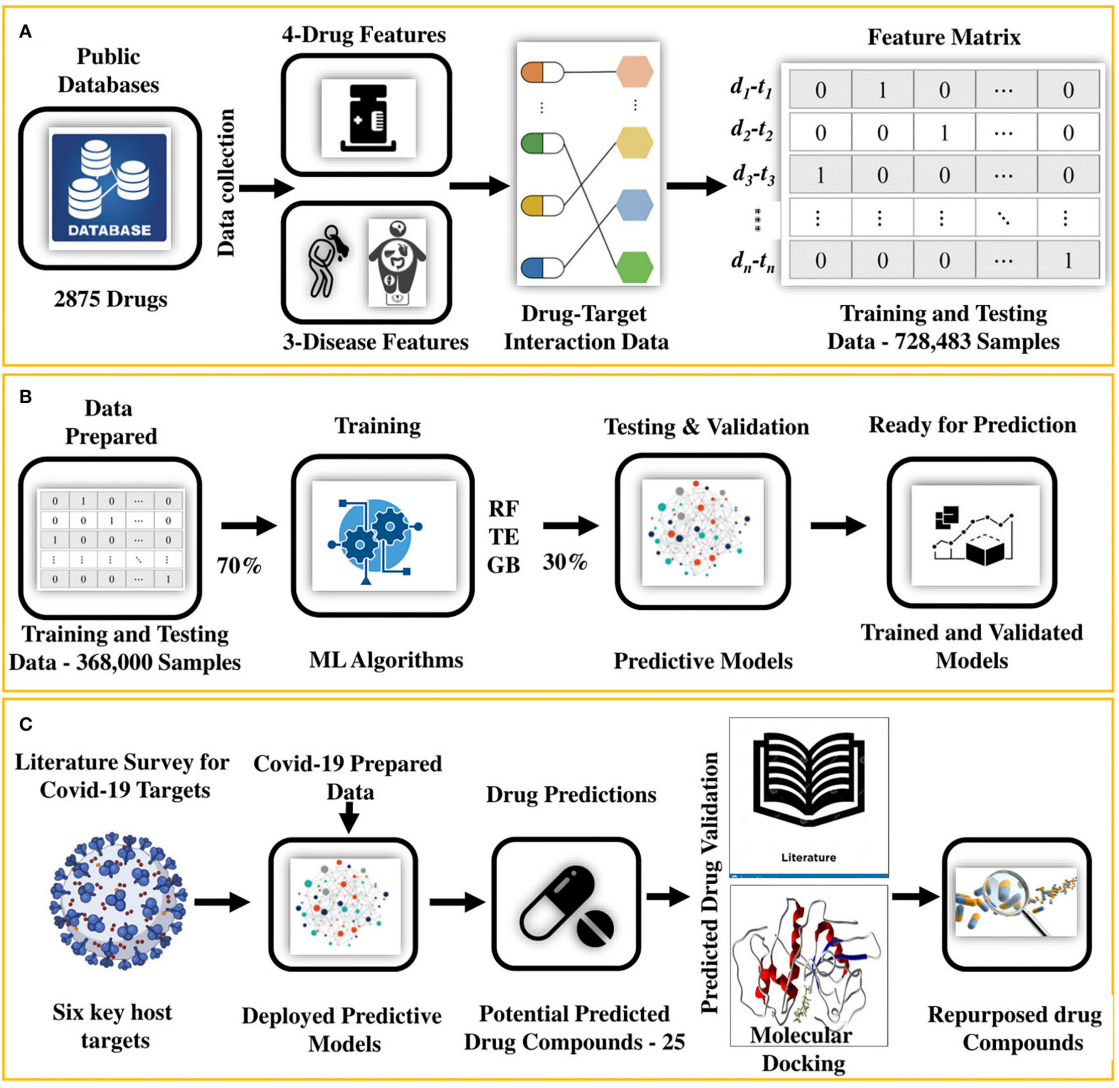
An integrated machine learning and molecular docking-based ensemble approach for drug repurposing. **(A)** It shows that the drug and disease data collected from the public databases are first arranged and then transformed into a feature matrix, **(B)** followed by training and testing of the predictive models (RF and TE). **(C)** After training and testing, the data for the COVID-19 host targets are prepared from the literature review and fed to the predictive model for getting the potential repurposed drugs. Potential hits are then validated by using literature mining and collecting the evidence in the form of papers, patents, and database evidence. For the remaining predictions, molecular docking is performed to find the binding affinity of the drugs. Finally, the drugs with the highest binding affinity are prioritized as the potential repurposed candidates ready for the preclinical and clinical tests.

In this study, we present an integrated machine learning and molecular docking-based drug repurposing framework that systematically uncovers undiscovered DTIs. This approach is based on the notion that (i) given known and unknown sets of DTI data enriched with various aspects of drug and disease features (Figure 1A), (ii) machine-learning models can be trained (Figure 1B). The trained models (iii) can be deployed to determine new DTI pairs for a given set of proteins functionally associated with diseases causing viral infection, such as COVID-19 (Figure 1C). We employed this approach along with (iv) the confirmation of the indications from the literature survey and (v) by performing molecular docking to further ensure the interactions (binding affinity) between predicted drug–target pairs as shown in Figure 1C. Following this approach, 25 potential drug candidates were predicted for COVID-19. Literature-based validation confirmed that 12 out of 25 (48%) drug compounds were already used in COVID-19. For the remaining 13 compounds, molecular docking was performed. Based on the docking results and prediction scores, four potential drugs were suggested to be pre-clinically and clinically validated for COVID-19.

## Materials and Methods

In this step, we followed the five steps shown in Figure 1 to identify the repurposed drugs for COVID-19 (drug repurposing).

### Dataset Preparation

The dataset contains four drug features (drug chemical structures, side effects, target sequences, and drug-targeted genes) and three disease features (disease gene sequences, disease specificity indices, and disease observable traits).

#### Chemical Structures

Chemical structures (40) in SMILES form have been used in many studies. Simplified molecular-input line-entry system (SMILES) is the form of a line notation for describing the structures of chemical species and SMILES was obtained from DrugBank (41), PubChem (42), and ChEMBL (43) during 2021–2022 using application programming interface (API) in KNIME as shown in Supplementary Figure 1. To make the data machine-learning ready, one-hot encoding (44, 45) was done to convert SMILES data into a 1,430-dimensional vector called the Drug-S Vector. The vectors were subsequently collected as the bit vector using the KNIME node called “Create Collection Column.”

#### Drug–Target Sequences and Genes

Each drug targets one or more proteins (18, 46) and receptors on proteins are the most prominent targets for drugs. In this study, the drug–target sequences were extracted from Uniprot and DrugBank during the academic year 2021–2022 (47). Initially, DrugBank IDs were mapped to Uniprot IDs followed by using API in KNIME to retrieve sequence data from Uniprot. The “Sequence Properties” node in KNIME is used to extract the properties, such as the number of positively and negatively charged residues, mol-weight, hydrophobicity, and aliphatic index. Finally, one-hot-encoding (48) was applied to extracted features and drug genes to transform features into the bit representation of the 2,294-dimensional vector.

#### Drug Side Effects

**Figure 2:**
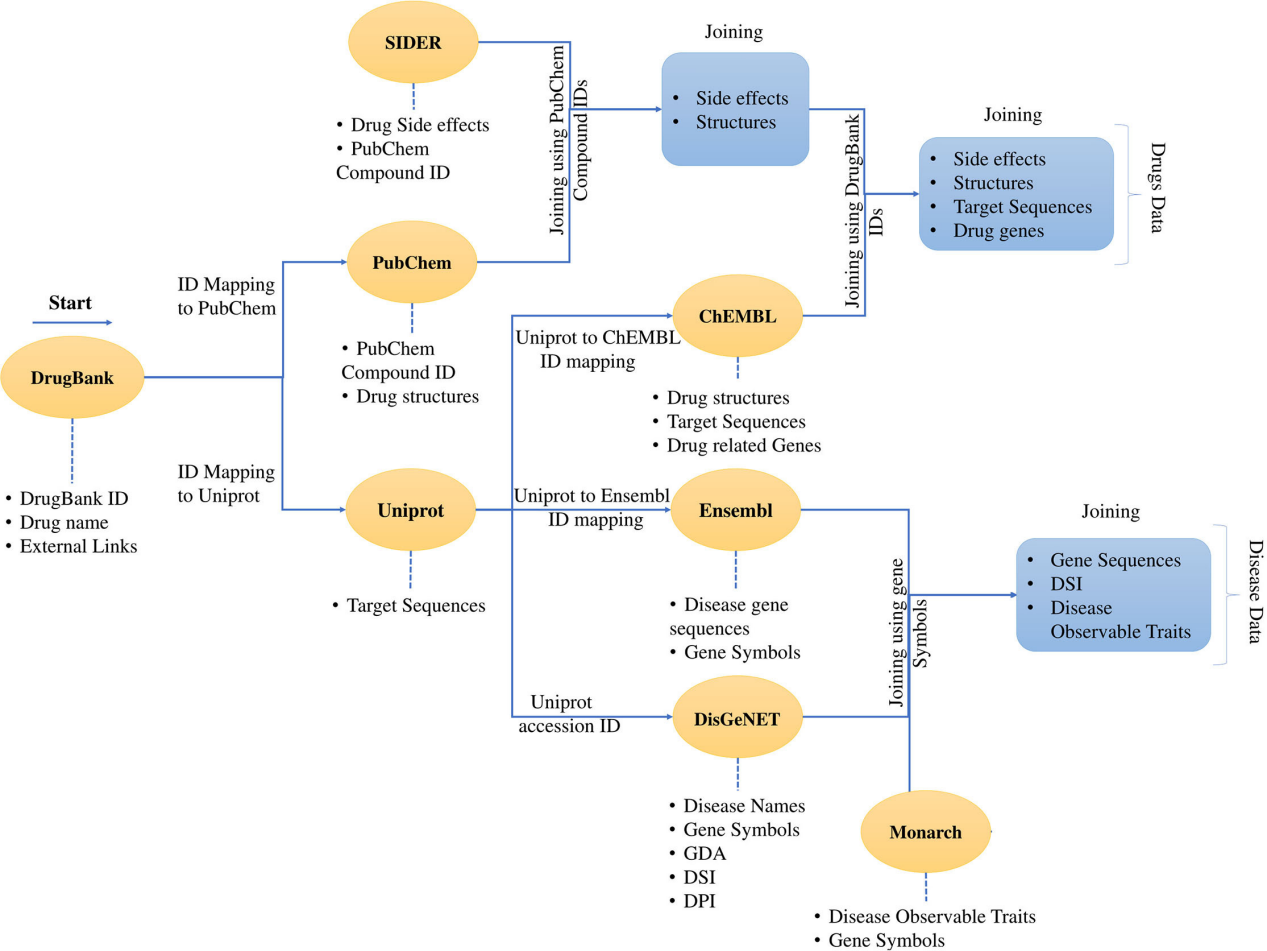
Process of data collection and data mapping. It shows the creation of a dataset and merging of different data sources using cross-database identifiers. The dataset contains the data for the drugs and the data for the diseases. The data download was started with DrugBank data with external links. Then the data was mapped to PubChem and Uniprot. It was followed by the mapping of the SIDER data using the PubChem IDs and the mapping continues through ChEMBL and all the four drug features are presented in a tabular form with identifiers from mentioned databases. The same process follows for the disease data, and finally, the disease data is also represented in the tabular form. The developed dataset contains the drug and disease-related data enriched with various features.

As a common hypothesis, drugs with similar side-effect profiles share similar therapeutic effects through a shared mechanism of action (49). The drug side-effect data of 1,430 FDA-approved drugs were downloaded from the SIDER database (27). SIDER provides mapping to PubChem which was used to map side effects to drug structures, protein sequences, and gene data, as shown in Figure 2. To make side-effect data machine-learning-ready, one-hot encoding was used in the KNIME analytics platform as shown in Supplementary Figure 2 resulting in a binary vector string of 5,868-dimension called Drug-Se.

#### Disease Gene Sequences and DSI

In recent years, there has been a tremendous increase in the knowledge accumulation of gene–disease associations. It is important to facilitate clinical practice using this knowledge (50). In our study, gene-disease associations (GDA) were collected from DisGeNET (51–53) that contains more than 400,000 GDA across all databases (54). Moreover, the Uniprot IDs were used along with gene symbols from DisGeNET to extract gene sequences from the Ensemble database (55, 56) using API in KNIME (Supplementary Figure 1). Drug sequences were converted using one-hot encoding into a binary string vector. Additionally, for the disease specificity index (DSI) (51), the vectors generated are referred to as Dis-Ge for one-hot-encoded gene sequences of 1,321 length. Whereas, the DSI values are left named as DSI.

#### Disease Observable Traits

Disease observable traits have been utilized for drug discovery and development (57–59). Drugs discovered based on phenotyping screens have surpassed the drug screened and developed through a molecular drug–target-based approach (60, 61). In this study, 10,881 human diseases with 8,662 phenotypes were retrieved from the Monarch database (62). The phenotypes were encoded using a one-hot encoder which produced vectors (Dis-Tr) of 5,212 lengths as shown in Table 1.

**Table 1:** Drug and disease features in the dataset created.

**Dataset**	**Drug features**				**Disease features**			**Dataset features**	
	**Drug structures**	**Drug–target sequences**	**Drug related genes**	**Drug side effects**	**Disease gene sequences**	**DSI**	**Disease observable traits**	**+ve**	**-ve**
	1,430	2,294	2,893	5,868	1,321	1,321	5,212	368,483	360,000

*The table mentions the total number of each feature in the dataset. It contains four drug features and three disease features. The details of the different features and their mapping process are discussed in detail in Section Dataset Preparation*.

#### Positive and Negative Samples

After collecting the known drug- and disease-related features data (DTIs), we assumed them to be positive samples, and the unknown interactions achieved through the randomized shuffling of the positive samples were assumed to be negative samples (63) followed by upsampling of the negative samples to create a balanced dataset. The rationale behind this is that conventional methods of unknown interactions between targeted drugs as negative examples may result in bias because unknown interactions between targeted drugs may contain undetected interactions between the targeted drugs. This was overcome by finding the duplication between the positive and negative samples and it was ensured that no pair from positive samples matches exactly to negative samples.

### Model Prediction Techniques

For bioinformatics research, machine learning plays a significant role in the filtering and comprehension of patterns in a given dataset (63). Our proposed study presents machine-learning models trained on various aspects of the drugs and disease data. The statistics for the datasets used in this study are provided in Table 1. The overall purpose of the workflow was to predict DTIs for COVID-19. The process can be divided into five steps. First, the models were trained using the training data, and second, the trained models were tested and cross-validated using the testing and cross-validation data, respectively. In this study, different machine-learning models were tested and evaluated. The ML models (algorithms) used in this work are Random Forest (RF), Tree Ensemble (TE), and Gradient Boosted Trees (GB).

#### Random Forest

It contains multiple individual decision trees that function as divisions. Every individual decision tree is bounded by predictions guided by the class, layer, and the soundest prediction results in the model. RF performs efficiently with large types of data elements from one decision tree, and its accuracy can be maintained even if the data contain missing values. In addition, the fair training time complexity expressed as O(n^*^log(n)^*^d^*^k) and excellent space complexity of the RF models given as *O(depth of tree* ^*^k) makes it a good choice in such applications. In the given time and space complexity notations, “*n”* shows the number of training examples, “*d”* shows the data dimensions, and “*k”* shows the number of models. In our work, we used the KNIME node “Random Forest Learner” with the parameters shown in Supplementary Table 1. The model (RF) in this study functions in two ways. First, it was trained, tested, and validated; then, the results from the model were used individually and combined or averaged with other models. Finally, predictions for the COVID-19 DTI data were obtained after training and testing.

#### Tree Ensemble

Similarly, we used the KNIME node “Tree Ensemble Learner” to develop the TE model. It contains an ensemble of decision trees. Typically, each tree in ensemble learning is developed using different sets of rows or columns. Whereas, rows are called records and columns are called attributes. The key idea behind ensemble learning is that a group of weak learners combine to form a strong learner. Moreover, like RF, the time complexity of the TE algorithm is fair, and it is represented by O(n^*^log(n)^*^d) and the space complexity of the TE is excellent. Similarly, in the given time and space complexity notations, “*n”* shows the number of training examples and “*d”* shows the data dimensions. In, this work, we used the “Tree Ensemble Learner” with the parameters listed in Supplementary Table 1. Like RF, TE learning algorithms also function in two ways. Additionally, the data prepared for COVID-19 were used to predict the DTIs to repurpose potential drugs for COVID-19.

#### Gradient Boosted Trees

Aiming at classification, it learns gradient boosted trees. It uses regression trees in a shallow form. Along with RF and TE, we used the “Gradient Boosted Trees Learner” node in KNIME for model development. The time and space complexity of the GB trees is also like the other two. Following the other two, GB was trained, tested, and validated, and the results from the model were combined and averaged with other models. After training and testing, predictions for the COVID-19 DTI data were obtained. The parameters used for the GB trees are also given in Supplementary Table 1.

### Training and Testing Procedure

The prepared dataset was split into three portions: training, testing, and validation using the train-test split method. For each of the classifiers (Section Model Prediction Techniques), 70% of the data were used as training data that were randomly drawn and contained both positive and negative samples. The remaining 30% of the data were used for performance testing (15%) and validation (15%).

While selecting the best models, we used a part of the training data and tuned the hyperparameters. This procedure was followed by the testing of the trained models with 15% of the data split for the testing purpose. The process was repeated to fine-tune the hyperparameters. Once the testing accuracy was sufficiently high, the validation data were used to perform the 10-fold cross-validation of the models. During this initial procedure, we eliminated GB because it did not perform appropriately, compared to RF and TE. Therefore, we developed and trained the models; the list of parameters is given in Supplementary Table 1. The models were subsequently run on the validation datasets to observe the predictive performance using the evaluation parameters (Section Model Evaluation Techniques) that testifies to the effectiveness of the training performed. At the end of the training, the best-performing models as per the Mathew correlation coefficient (MCC) and other important parameters were selected. Finally, the models were either used in the combination (averaging the output of the models) or only the RF.

### Model Evaluation Techniques

The different parameters used to evaluate the machine-learning model are discussed below.

#### Accuracy

This is the ability of a classifier to differentiate between positive and negative samples correctly. To determine the accuracy, true positive and true negative should be known across all cases. The accuracy can be measured using Equation (i).


(i) $$\begin{array}{l} Accuracy=\;\frac{TP+TN}{TP+TN+FP+FN} \end{array}$$

Here, TP is true positive, TN is true negative, FP is false positive, and FN is false negative.

#### Precision and Recall

Precision refers to the correct ratio of positive reactions, whereas recall shows the ratio of positive samples that have been predicted correctly. Precision can be calculated as per Equation (ii), and recall can be calculated according to Equation (iii).


(ii) $$\begin{array}{lll} Precision & = & \frac{TP}{TP+FP} \end{array}$$

(iii) $$\begin{array}{lll} Recall & = & \frac{TP}{TP+FN} \end{array}$$

#### F1 Score

This measures the balance between precision (p) and recall in the system. Equation (iv) was used to calculate the precision.


(iv) $$\begin{array}{l} F1\;Score=\;\frac{2*\left[\;Precision*Recall\;\right]}{\left[\;Precision+Recall\;\right]} \end{array}$$

#### Mathew Correlation Coefficient

The value of MCC ranges from −1 to 1. In this range, −1 is a zero-credible binary-learning method, whereas 1 is a completely confident binary-learning method. The calculation formula for MCC is given by Equation (v).


(v) $$mcc=\;\frac{TP*TN-FP*FN}{\sqrt{\left((TP+FN\right)}*\left(TN+FP\right)*\left(TP+FP\right)*(TN+FN))}$$

### Prediction Validation Techniques

After the training, testing, and validation, the models were deployed. The data for COVID-19 host targets were collected from the literature. The information sources of the collected host targets for COVID-19 are given in **Table 3** along with other information. The data were pre-processed, and the predictions were obtained from the deployed ML models. The predictions (new DTI) were validated in the following two ways.

#### Literature-Based Validation of the New DTI Predictions

After the new DTI predictions were obtained for the COVID-19 targets, we performed a manual literature search. We used combination models (RF and TE) and individuals, and for both types of models, literature-based evaluation was performed (64, 65). As a result, we labeled the new predicted DTIs valid when at least one publication or database evidence showed the use of the predicted drug in COVID-19. This can be further divided into two categories: first, the evidence from the publications that could mention either the computational approach or a combination of the computational approach along with wet-lab experiments, and second, the database evidence. The databases, such as DrugBank, mention drugs along with their indications and are updated periodically.

#### Molecular Docking

After initial validation based on the literature, molecular docking was performed using AutoDock Vina for the unconfirmed DTIs. Moreover, for docking experiments, the co-crystallized structures of the host COVID-19 targets were downloaded from the Protein Data Bank (RCPDB) along with their PDB IDs. PyMol (66, 67) visualization software was used to visualize the target protein and extract the binding sites after deleting the water molecules and removing the backbone ribbon structure of the protein. Additionally, all the ligand molecules were also removed. The prepared protein structures were later used as inputs to Auto Dock Vina (67) for molecular docking, where hydrogen atoms and Gasteiger charges were added to the structures.

For the ligands, 3D structures were downloaded from PubChem (68) in structured data format (SDF). The structures were first converted into Protein Databank (PDB) format in PyMol, followed by structure preparation in AutoDock Vina. The prepared ligand structures were named PDBQT format. Before performing docking, binding pockets were created using the known binding sites in the crystal structure, and docking was run for 10,000 iterations. The results for each ligand–target docking were collected separately and analyzed for binding affinity values. Finally, ligands with higher binding affinity values were prioritized, and then the re-docking procedure was carried out in AutoDock Vina with 10,000 iterations. Based on the docking and re-docking results, comparison potential COVID-19 drugs were suggested for preclinical and clinical validation.

## Results and Discussion

Here, we discuss the results obtained from our proposed machine-learning-based prediction models (SperoPredictor®) for DTI implementation on the generated drug–disease (DTI) dataset, followed by novel DTI prediction for COVID-19, their literature-based validation, and molecular docking-based validation.

### SperoPredictor Training, Testing, and Validation

**Figure 3:**
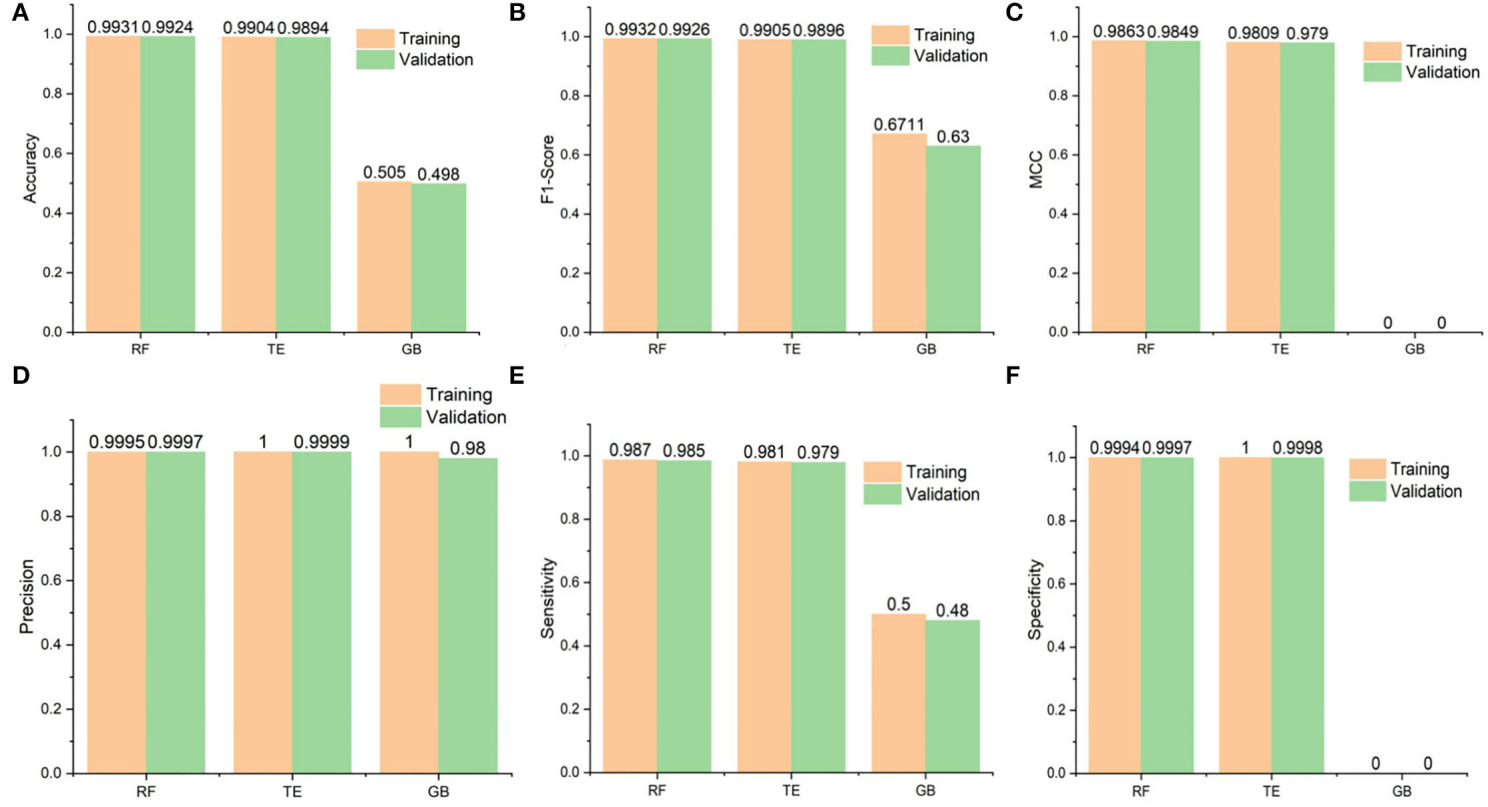
Performance statistics of the algorithms used in the study. Additionally, it shows the training and 10-fold cross-validation results of the Random Forest (RF), Tree Ensemble (TE), and Gradient Boosted (GB) classifiers. **(A)** Shows the accuracy results of the classifiers and RF seems to perform best followed by the Tree Ensembl predictor, **(B)** in case of F1 scores, RF again performs better than the rest whereas the GB performance is worst, **(C)** whereas MCC values are also best for the RF but **(D)** Precision results are best for the TE during training and validation followed by the RF and GB, finally **(E,F)** for the Sensitivity and specificity, respectively, RF performs best and GB is constantly worst.

In our work, we handled the DTI prediction for COVID-19 as a binary classification problem. The developed BioSpero's SperoPredictor® accepts the drug–disease data in a one-hot-encoded format and performs binary predictions between drugs and targets as active (valid or interacting) or inactive (invalid or non-interacting) for a given set of protein targets. The predictions were further validated using literature-based evidence and molecular docking, as shown in Figure 1. Moreover, to train the models, we generated a multiple feature-based dataset for DTIs from multiple databases. The data collection for 1,430 drugs and 2,265 protein targets (Table 1) was partially performed through an application programming interface (API) in the KNIME analytics platform, and subsequently processed and transformed using KNIME nodes. API automatically extracts the data from databases using a set of identifiers and the data-mapping process is shown in Figure 2. To validate the models, we split the dataset into training (70%), testing (15%), and validation (15%) datasets. Moreover, 10-fold cross-validation was performed on the tested models, and the performance statistics are shown in Figure 3. The results are further summarized in Supplementary Table 2, illustrating the accuracy, MCC, F1 score, precision, sensitivity, and specificity, whereas the corresponding results are shown in Figure 3. The given parameters were calculated during testing and cross-validation.

The accuracies of the RF model during testing and validation were 0.9931 and 0.9924, respectively. Similarly, the TE accuracies remained at 0.9904 and 0.9894, respectively, during the testing and validation of the model. In addition, the precision-recall values are also presented for the proposed models. It is important to mention that precision is also referred to as positive predictive value (PPV) and recall (sensitivity) is referred to as the true positive rate (TPR) (63); specificity, also called true negative rate (TNR), is also mentioned in Supplementary Table 2. Higher values for precision, recall, and specificity indicate the greater predictive performance of the trained models.

### Comparison With the Existing Methods

In this section, we provide a comparison between SperoPredictor and three recent state-of-the-art methods. The first method proposed efficient machine-learning methods with a case study on COVID-19 (63). In these studies, SVM, RF, XGBOOST, and DBN were used. DTIs were predicted using RF and SVM for COVID-19. They predicted drugs for ACE2 with 100% confidence. Another method is based on the deep learning structure model (CNN) to gain the sequences (Amino acid) in 1D representation. According to the results, the use of CNN for data representation improved the performance, compared with traditional methods, and the performance among all models is quite visible. The other method offers a novel approach to developing negative DTI. In this study, many lasso models were used to combine multiple sets of features to examine the prediction power and DITs (69). Lasso DN's suggested comparing the performance to Lasso, support vector machine (SVM), standard logistic regression (SLG), and DNN models. Additionally, another study proposed DTI prediction using Lasso with RF. FP2 and PsePSSM fingerprints were used for feature extraction, followed by the removal of redundant information (70). Our method performs better than all the other methods, achieving the best results in testing and cross-validation, as shown in Table 2. As shown in Figure 3 and Table 2, the highest accuracy achieved in our work is 0.9931 (99.31%) in the case of RF and 0.9904 (99.04%) in the case of the TE algorithm which is approximately 5% higher than the first (63), 6% higher than the second (69), and 7% higher than the third (70). Similarly, when the results are compared for other parameters, such as MCC, our proposed models exhibit better performance at 0.9863 for RF and 0.9809 for TE as compared to other studies done in (63, 69, 70). Another recent study done in (71) uses SVM and RF models to repurpose the drugs for COVID-19. The accuracies of the classifiers as shown in Table 2 are 0.90 and 0.82, respectively, outperformed by RF and TE models presented in our study.

**Table 2:** Model performance comparison between related and our work.

**Study**	**The methods**	**Accuracy**	**MCC**
Efficient machine-learning model (63)	RF	0.947	0.945
	SVM	0.93	0.917
Lasso-DNN method (69)	SVM	0.81	-
	ANN	0.9277	-
Lasso with random forest (70)	RF	0.9809	-
Repurposed drugs for COVID-19 using AI and ML (71)	RF	0.82	-
	SVM	0.90	-
Our proposed work	RF	0.9931	0.9863
	TE	0.9904	0.9809

*The models from the related were listed along with their performance parameters. The accuracy and MCC of the Random Forest and Tree ensemble classifiers were matched with the state-of-the-art methods from the literature*.

### Application in COVID-19

*De novo* drug development takes 12–15 years with a $2–3 billion investment. Drug repurposing has emerged as an effective alternative solution to respond in case of epidemics and pandemics. In this regard, repurposing potential antiviral drugs for COVID-19 is the only solution to counter the sudden emergence of pandemics. Moreover, the recurrence of COVID-19 is significantly affected by the response of the human immune system. To date, there has been extensive research conducted involving different methods (such as the data-driven method) on COVID-19. These methods use different resources for data collection and analysis, such as DrugBank, TTD, STITCH, and ZINC. Additionally, antiviral drugs targeting COVID-19 can be placed in one of two categories. First, antiviral drugs target the host targets of the virus to impede the aggregation of the virus, and second, drugs boost the immune response over a wide spectrum (72).

Moreover, a study using the deep learning model (73) conducted pre-training on the interactions, and MT-DTI identified the EGFR receptor successfully as the drug–target that has been associated with 30 candidates out of 1,094 from the DrugBank. This study did not require 3D structural information to predict the interactions between drugs and targets. However, there is no evidence to support the positive action of drugs in COVID-19 (74). The drug Atazanavir exhibited reasonable efficacy and binding affinity toward COVID-19-target proteins. In addition, the FDA approved Remdesivir for use in patients aged 12 years and above (75). Another study performed drug repurposing for COVID-19 using a literature-based approach (76). They used a scientific approach called the literature-based discovery (LBD) and compared it with three other similar models. They concluded that semantic models are most suitable for drug repurposing.

A recent study (77) suggests the application of AI to accelerate the drug repurposing process (78). This further demonstrates the importance of different AI approaches and discusses the different drugs that are currently under clinical trials in various phases. Similarly, another study discusses how AI models are used in precision medicine and how AI models can accelerate drug repurposing in COVID-19. Furthermore, AI along with network-based approaches, can be a powerful and innovative alternative to drug repurposing. According to these reviews, AI-based tools can be used to reposition drugs for other human-related diseases, focusing on COVID-19.

Here, we focused on predicting DTIs using SperoPredictor, the proposed machine learning and molecular docking repurposing framework that was trained on drug–disease-enriched data (the corresponding statistics are shown in Table 1). The data were collected from multiple sources and mapped to a single table using the cross-database identifiers and the mapping process is shown in Figure 2. In our study, to predict the repositioning drugs for COVID-19, we deployed our trained models with high accuracy and specificity of 99.3–99.94% (RF) and 99.0–99.98% (TE), respectively.

To get the predictions for potential repurposed drugs for COVID-19, a literature survey was conducted, and the key host-target proteins of COVID-19 were collected. The target proteins are shown in Table 3. After collecting the targets, the data, including the observable traits, Ensembl gene ID, and DSI index, for these targets were prepared, as shown in Supplementary Table 5. The observable traits were collected from the Monarch database, whereas gene IDs and DSI indices were collected from the Ensemble database. The data were subsequently fed into the workflow for processing and gene sequence extraction, and the workflow is shown in Supplementary Figure 3. Following the processing and transformation of the COVID-19 target data, they were provided as input to the deployed RF and TE model for the predictions, as shown in Supplementary Figure 4. The models were used in two configurations: first, the combination of both models was used, and as a result, two drugs were predicted; second, the RF model was used, and a total of 25 drugs were predicted.

**Table 3:** Key host-target proteins in COVID-19.

	**Uniprot**	**Entry name**	**Protein name**	**PDB ID**	**References**
1	O15393	TMPS2_HUMAN	Transmembrane protease serine 2	7MEQ	(79, 80)
2	P09958	FURIN_HUMAN	Furin	5MIM	(81)
3	Q9BYF1	ACE2_HUMAN	Angiotensin-converting enzyme 2	7V8V	(82)
4	Q2M2I8	AAK1_HUMAN	AP2-associated protein kinase 1	5L4Q	(83)
5	O14976	GAK_HUMAN	Cyclin-G-associated kinase	4O38	(83)
6	P07711	CATL1_HUMAN	Procathepsin L	1CS8	(84)

*The Uniprot identifiers for each target are mentioned along with their entry names and protein names. PDB IDs are also provided which are later used in molecular docking also. Additionally, references for each protein target are given*.

Our strategy for validating the prediction was based on literature-supported evidence and molecular docking. Two drugs predicted from combination models (RF and TE) were validated from the literature, and evidence suggested their repurposed use in COVID-19. Whereas 12 out of 25 drugs predicted using RF were found in the literature, suggesting their use in COVID-19. This means 48% of the predicted drugs were known to have anti-COVID-19 activity. All predicted drugs, along with evidence found in the literature, are specified in Table 4 with prediction confidence and literature evidence. Whereas, all 25 predicted drugs, along with the information, are specified in Supplementary Table 3.

**Table 4:** Drugs predicted for COVID-19 with their prediction probability and reference links.

	**DrugBank ID**	**Drug name**	**Targets**	**Prediction Confidence**	**References**
1	DB01054	Nitrendipine	AAK1	0.909	(85)
2	DB12610	Ebselen	FURIN AAK1	0.916	(86, 87)
3	DB04954	Tecadenoson	AAK1	0.912	(88)
4	DB12831	Gabexate	ACE2 CTSL	0.912	(89)
5	DB12945	Dihydralazine	TMPRSS2 CTSL ACE2	0.946	(90)
6	DB13014	Hypericin	AAK1	0.901	(91)
7	DB13025	Tiapride	FURIN AAK1	0.917	(92)
8	DB13132	Artemisinin	CTSL ACE2	0.964	(93, 94)
9	DB13141	Ambroxol acefyllinate	TMPRSS2 CTSL ACE2	0.94	(95, 96)
10	DB13620	Potassium gluconate	AAK1	0.911	(97, 98)
11	DB13875	Harmaline	GAK FURIN ACE2	0.943	(99, 100)
12	DB13876	Brofaromine	TMPRSS2 CTSL ACE2	0.948	(101)

*The mentioned drugs are the results of the drugs from the Random Forest model and model combination (Random Forest + Tree Ensembl). The first two drugs are the result of the combination models. Moreover, the table contains the drugs and their DrugBank IDs which are validated from the literature for their use in COVID-19. It supports and adds to the credibility of our developed ML models. Additionally, the COVID-19 targets in the form of Uniprot IDs along with prediction confidence and literature-based evidence*.

### Molecular Docking and Re-docking for Binding Affinity Prediction of Compounds Against SARS-CoV-2 Host Targets

**Figure 4:**
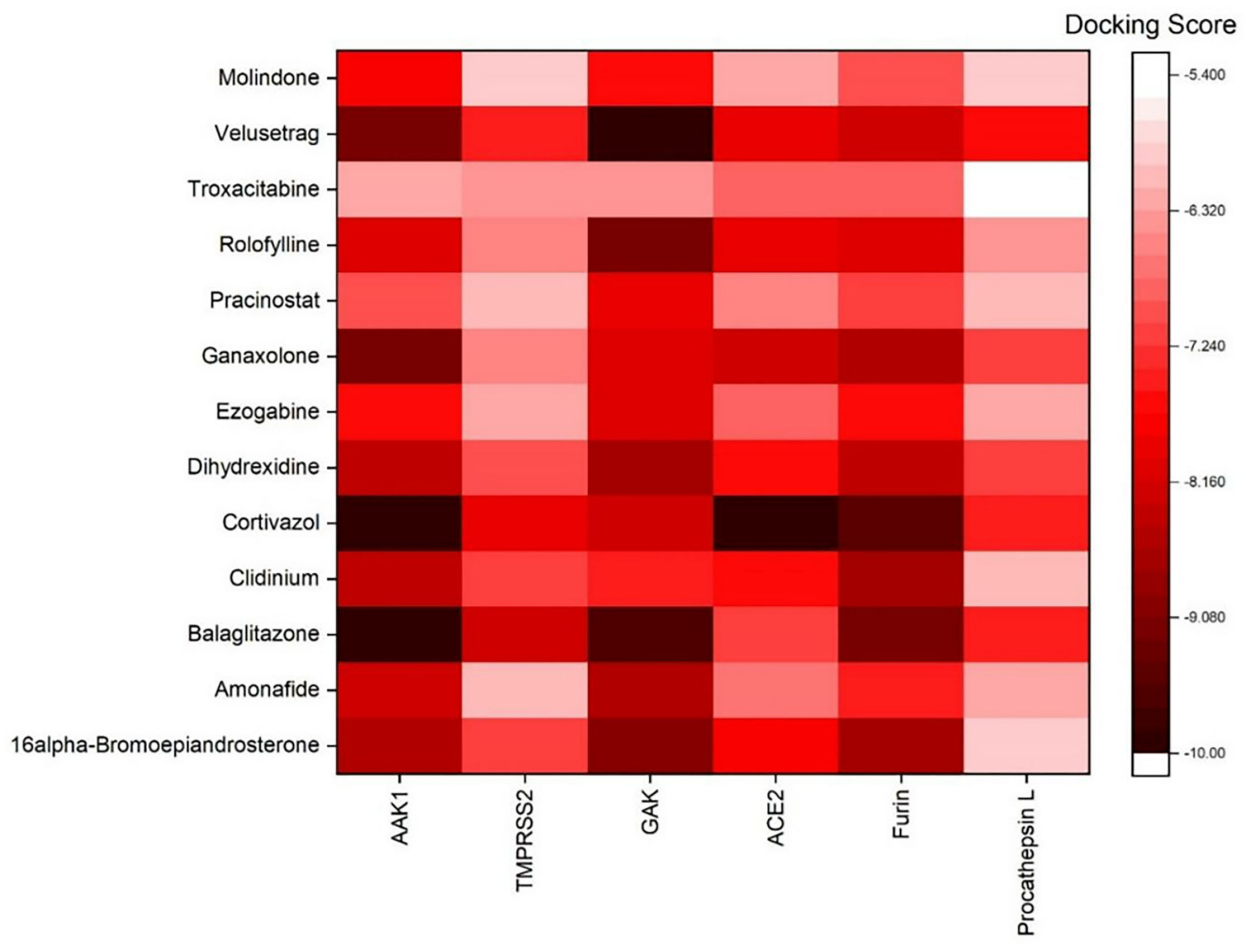
Binding affinity or docking scores of the predicted repurposed drugs against six COVID-19 host proteins. The candidate predicted drugs are mentioned along the y-axis whereas the host COVID-19 protein targets are mentioned along the x-axis. The Docking score bar is showing the maximum to the minimum value, where the dark red color presents the maximum score of −10 and the white fed color shows −5.4. The highest binding affinities of 9.0 to −10.0 kcal/mol were achieved for Velusetrag-GAK, Cortivazol-ACE2, Balaglitazone-AAK, and Cortivazol-ACE2 complexes.

AutoDock Vina was used to perform molecular docking between the predicted ligands (for SARS-CoV-2) and SARS-CoV-2 host protein targets. All the selected host COVID-19 targets are listed in Table 3. The binding sites and PDB IDs of the selected proteins are shown in Supplementary Table 4. Ligand structures were downloaded in 3D SDF format from PubChem and converted to the PDB format using PyMol software. The preparation of 13 ligands (not confirmed in the literature) and six targets was conducted in the AutoDock Vina environment. Each ligand molecule was docked against all the six selected host COVID-19 protein targets. The binding affinity results are shown in Figure 4. As a result of molecular docking, the lowest binding affinity score was found as −5.4 (kcal/mol), and the highest score was −10 (kcal/mol). The top-scoring drugs for Adaptor Protein 2-Associated Kinase 1 (AAK1) with binding affinities ranging from −9.0 to −10.0 kcal/mol were Balaglitzone (ΔG = −9.9 kcal/mol), Cortivazol (ΔG = −9.9 kcal/mol), Ganaxolone (ΔG = −9.1 kcal/mol), and Velusetrag (ΔG = −9.1 kcal/mol) (Supplementary Table 6). Similarly, for Cyclin-G-Associated Kinase (GAK), the highest-scoring drugs were Balaglitzone (ΔG = −9.6 kcal/mol), Velusetrag (ΔG = −10.0 kcal/mol), 16-alpha Bromoepiandrosterone (ΔG = −9.0 kcal/mol), and Rolofylline (ΔG = −9.1 kcal/mol) (Supplementary Table 6). For the Angiotensin-Converting Enzyme 2 (ACE2), Cortivazol (ΔG = −10.0 kcal/mol) performed the best and for Furin, Cortivazol (ΔG = −9.4 kcal/mol), Balaglitzone (ΔG = −9.1 kcal/mol), and 16-alpha Bromoepiandrosterone (ΔG = −8.7 kcal/mol) demonstrated the best results (Supplementary Table 6). Additionally, for the Transmembrane Protease Serine 2 (TMPRSS2) and Procathepsin L, the docking score of all the drugs was found to be below −8.0 kcal/mol, as shown in Figure 4. To further validate the top hit (six) drugs, re-docking was performed and results were found consistent as shown in Supplementary Table 6. Finally, the top hit predicted drugs interacting with high-binding affinity to at least two host targets based on the molecular docking score were analyzed and suggested as an effective treatment to be pre-clinically and clinically validated for COVID-19 (Table 5).

**Table 5:** Finally suggested four drug candidates for COVID-19 with their target information.

	**Drug bank ID**	**Drug name**	**COVID-19 targets**	**Free energy of binding** **(Kcal/mol)**	**Free energy of binding (Kcal/mol) Re-docking**	**Prediction score**
1	DB13003	Cortivazol 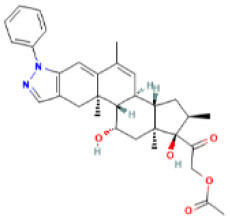	Adaptor protein 2 associated kinase 1 (AAK1)	−9.9	−10.0	0.94
			Angiotensin-converting enzyme 2 (ACE2)	−10	−10.0	
			Furin	−9.4	−9.4	
2	DB12702	Velusetrag 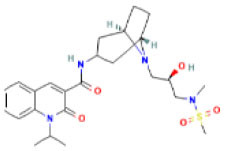	Adaptor protein 2 associated kinase 1 (AAK1)	−9.1	−8.6	0.944
			Cyclin-G-associated kinase (GAK)	−10	−10	
3	DB05107	16-alpha Bromoepiandrosterone 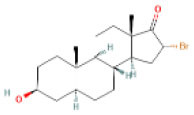	Cyclin-G-associated kinase (GAK)	−9.0	−9.0	0.902
			Furin	−8.7	−8.7	
4	DB12781	Balaglitazone 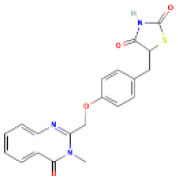	Adaptor protein 2 associated kinase 1 (AAK1)	−9.9	−10.1	0.946
			Cyclin-G-associated kinase (GAK)	−9.6	−9.7	
			Furin	−9.1	−9.1	

*Docking and pre-docking scores in kcal/mol are given which confirm the docking results as valid. Additionally, the prediction results of the drugs from SperoPredictor are also shown here*.

**Figure 5:**
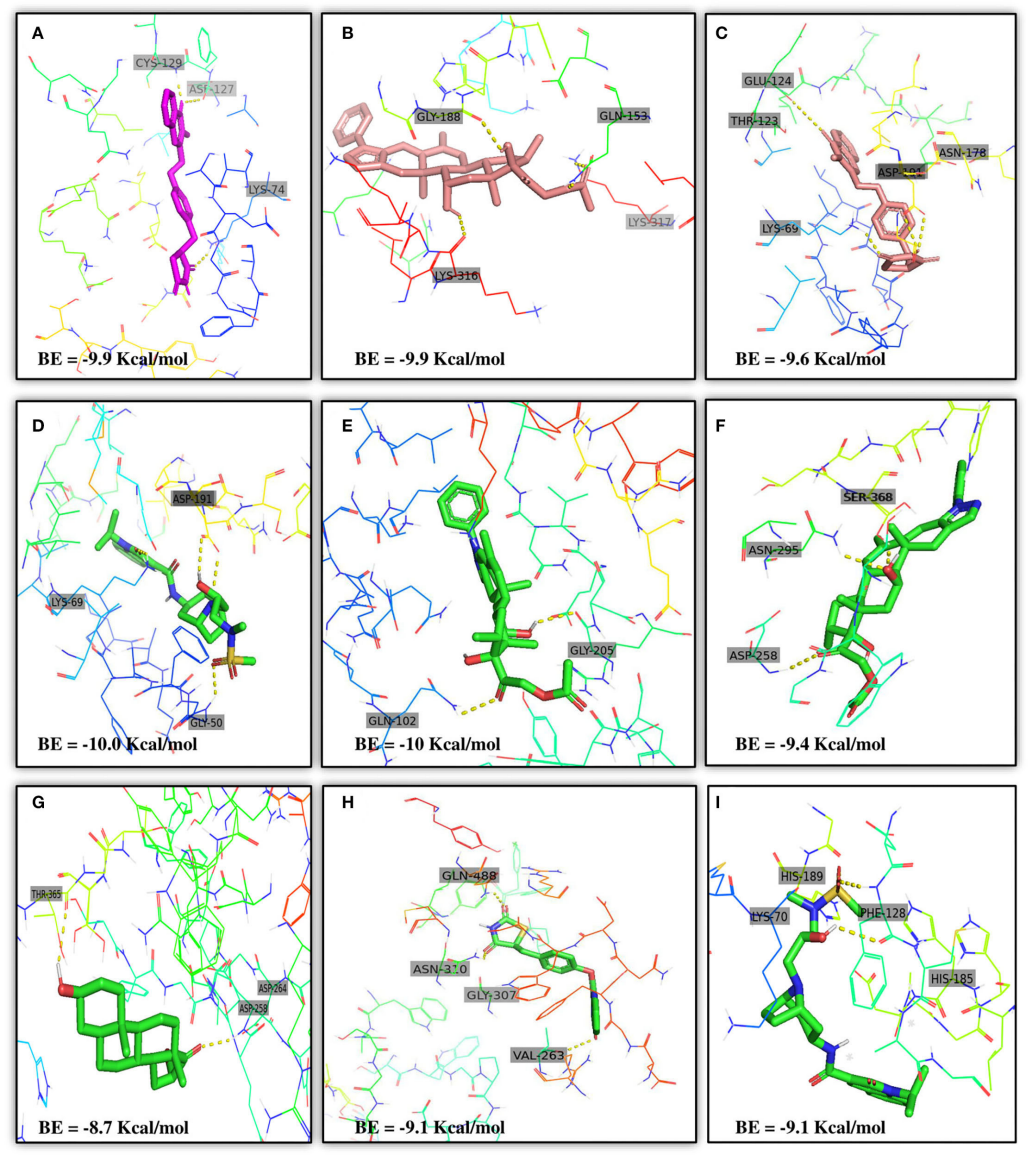
Results of the molecular docking of the potential predicted drugs. Further, it shows the molecular interaction, residues, and scores. **(A,B)** It shows the interaction of Adaptor Protein 2-Associated Kinase 1 (AAK1) with Balaglitazone, Cortivazol, and Velusetrag, respectively. The docking score of the small molecules is −9.9 for the first two and −19.1 for Velusetrag which suggests strong binding affinity during the interaction. Additionally, in **(C,D)** interaction of Cyclin-G-associated kinase with Balaglitazone and Velusetrag is shown, respectively, with docking scores of −9.6 and −10.0. Whereas **(E)**, shows the molecular interaction between Angiotensin-converting enzyme 2 (ACE2) and Cortivazol with a docking score of −10 followed by **(F–H)** Cortivazol, 16 alpha-Bromoepiandrosterone, and Balaglitazone results with Furin. The docking score for Furin-Cortivazol complex is −9.4, for Furin-16 alpha-Bromoepiandrosterone, the docking score is −8.7, and for Furin-Balaglitazone, the docking score is −9.1. **(I)** Cortivazol binding affinity with Adaptor Protein 2-Associated Kinase 1 (AAK1).

Moreover, the molecular interaction of the top four hit molecules (four drugs) interacting with at least two host COVID-19 targets based on the molecular docking scores are shown in Figure 5. This indicates the binding interaction along with the different host protein targets, their residues, and interaction scores highlighted in Figure 5. Based on the docking scores, small-molecule drugs (Table 6), such as Balaglitazone, are suggested for preclinical validation against Adaptor Protein 2-Associated Kinase 1 (AAK1) (Figure 5A), Cyclin-G-Associated Kinase (GAK) (Figure 5C), and Furin (Figure 5H). Similarly, the other top-ranked candidate drug Cortivazol is recommended against host COVID-19 targets, such as Adaptor Protein 2-Associated Kinase 1 (AAK1), Furin, and Angiotensin-Converting Enzyme 2 (ACE2), followed by Velusetrag, recommended for Cyclin-G-Associated Kinase (GAK) (Figure 5D), and Adaptor Protein 2-Associated Kinase 1 (AAK1) (Figure 5I); 16 alpha Bromoepiandrosterone is suggested for Furin (Figure 5G) as well as for Cyclin-G-Associated Kinase (GAK).

**Table 6:** Prioritized drugs with their original indication and host COVID-19 targets for which these drugs should be preclinically validated.

	**Drug name**	**DrugBank ID**	**Original indication**	**Repurposed for**	**Host COVID-19 targets**
1	Cortivazol	DB13003	Cluster headache- investigational (NCT00804895)	COVID-19	Adaptor protein 2 associated kinase 1 (AAK1)
					Angiotensin-converting enzyme 2 (ACE2)
					Furin
2	Velusetrag	DB12702	Gastroparesis and Alzheimer's-investigational	COVID-19	Adaptor protein 2 associated kinase 1 (AAK1)
					Cyclin-G-associated kinase (GAK)
3	16-alpha Bromoepiandrosterone	DB05107	Non-productive inflammation	COVID-19	Cyclin-G-associated kinase (GAK)
					Furin
4	Balaglitazone	DB12781	Diabetes mellitus, type 2–investigational	COVID-19	Adaptor protein 2 associated kinase 1 (AAK1)
					Cyclin-G-associated kinase (GAK)
					Furin

Furthermore, looking at the severity and state of emergency COVID-19 has caused, there is a dire need for effective treatments. One way to respond to the urgent need is to use Biotechnology platforms to swiftly repurpose the potential drug candidates. The emergence and re-emergence of pandemics emphasize the need of building accurate and robust drug repurposing platforms like SperoPredictor to make the process of drug discovery smooth and faster. Moreover, the proposed work offers many benefits, such as it contains ML models trained on drug–disease data enriched with multiple features. Drug data were enriched with four features, however, disease data were enriched with three features. Rigorously trained ML models were tested, cross-validated, and can be applied to any disease, such as was deployed for COVID-19 to repurpose the drugs with high accuracy. Despite the highly accurate and confident prediction results limitations, such as lack of data (1,430 Drugs) and preclinical validation models, are acknowledged and will be taken as a potential future direction that will enhance the effectiveness of the SperoPredictor. Since the trained machine-learning models yield high testing and validation accuracies (RF = 99.3 % and TE = 99.04 %) and the predictions were confirmed from the literature (12/25–48%), these drugs cannot be directly used in clinical trials. Currently, this study excludes the preclinical and clinical validation of the prioritized drugs. Most importantly, the limitation of the available data for drugs and disease is by far the major limitation of this study followed by the lack of negative data samples (which were later upsampled) which could lead to machine-learning models resulting in a high false-positive rate. Additionally, the validation of the remaining (not confirmed from literature) DTI was performed using the molecular docking approach. A lot of research work has been done and molecular docking approaches are used for the prioritization of the anti-COVID-19 drug compounds, still these approaches are not perfect. To overcome the uncertainty and improve the confidence in the results, we used the docking in two steps: first docking of the remaining drugs (13) followed by the prioritization based on the docking scores as shown in Supplementary Table 6 (6/13 were prioritized). Second, re-docking for the shortlisting of prioritized drugs was done and the results are shown in Supplementary Table 6. Finally, the four drugs were prioritized based on the number of COVID-19 targets and docking results. Each drug should have more than one target as shown in Table 6.

## Conclusion

The past and present efforts are focused on accelerating the drug development process through an alternative approach called drug repurposing. In this study, we present SperoPredictor, a machine learning and molecular docking-based repurposing framework with a use case in DTI prediction for COVID-19. The RF (99.3%) and TE (99.03%) can classify the DTIs with high accuracy. SperoPredictor, is a generalized framework that can be deployed as a rapid response. Additionally, most previous methods have focused on the features of proteins and sequences, involving the physical and chemical properties of drugs. The current proposed data integration method that accommodates the various aspects of drugs and diseases makes the predictions more confident. Similarly, a simple data pre-processing method saves running and processing time and space complexity. Moreover, using SperoPredictor models, we predicted 25 drugs (repurposed) for the SARS-CoV-19 host targets. According to the literature, 12 (48%) of the predicted drugs (25 drugs) have already been tested for SARS-CoV-19. Among the remaining 13 drugs based on molecular docking, re-docking and prediction confidence results of four drugs showing strong binding affinity are suggested for use in COVID-19. These drugs are Balaglitazone, Cortivazol, Velusetrag, and 16-alpha Bromoepiandrosterone. However, the majority of the predictions are validated by various literature sources and two-tier molecular docking validation is also performed. Thus, all the recommended drugs must be validated in various COVID-19 assays and clinical trials before being used in patients. We acknowledge the limitations in this study which will be taken as potential future directions and work along with possible drug combinations. This study is limited to the computational approach and excludes the *in vitro* validation. Additional limitations of this study include a limited number of drugs (1,430 drugs) and corresponding drug–target (2,294 targets) data along with the lack of negative data samples. Whereas, a higher number of drugs could contain and result in more potential anti-COVID-19 drugs and balanced data samples could result in better classification. Finally, the analysis of the novel DTI prediction for COVID-19 indicated that our approach could infer a list of novel DTIs that are practically applicable for drug repurposing. Moreover, the proposed approach can be used to repurpose the drug for any disease of interest following the same approach and this method can be applied in other ways to find the alternative uses for the existing drugs. However, in the future, the efficiency of this approach can be further enhanced by adding more data to train the models and using some state-of-the-art deep learning methods. In conclusion, predictive results of the SperoPredictor supported by literature evidence (12/25–48%) and good docking results between repurposed drugs (ligands) and SARS-CoV-19 host targets prove that our proposed models successfully identify potential repurposed candidates for COVID-19 treatment with high accuracy and confidence.

## Data Availability Statement

The original contributions presented in the study are included in the article/Supplementary Material, further inquiries can be directed to the corresponding author.

## Author Contributions

FA contributed to methodology, conceptualization, formal analysis, investigation, validation, writing the original draft, molecular docking, and machine learning. JLe and AS contributed to methodology, formal analysis, investigation, and validation. YK, IK, and FM contributed to formal analysis, investigation, and validation. KK contributed to conceptualization, formal analysis, validation, and writing, reviewing, and editing the manuscript. JLi contributed to formal analysis and validation. KC contributed to conceptualization, investigation, resources, supervision, and funding acquisition. All authors contributed to the article and approved the submitted version.

## Funding

This research was supported by the Technology Innovation Program (or Industrial Strategic Technology Development Program-Development of disease models based on 3D microenvironmental platform mimicking multiple organs and evaluation of drug efficacy) (20008413) funded by the Ministry of Trade, Industry and Energy (MOTIE, Korea).

## Conflict of Interest

JL and YK were employed by BioSpero. KC is owner of BioSpero. The remaining authors declare that the research was conducted in the absence of any commercial or financial relationships that could be construed as a conflict of interest.

## Publisher's Note

All claims expressed in this article are solely those of the authors and do not necessarily represent those of their affiliated organizations, or those of the publisher, the editors and the reviewers. Any product that may be evaluated in this article, or claim that may be made by its manufacturer, is not guaranteed or endorsed by the publisher.
