# Supplementary material for: SperoPredictor: An Integrated Machine Learning and Molecular Docking-Based Drug Repurposing Framework With Use Case of COVID-19
Source: Front Public Health. 2022 Jun 16;10:902123. doi: 10.3389/fpubh.2022.902123 (PMC9244710; doi:10.3389/fpubh.2022.902123)
Supplement: Supplementary file 1 [file Data_Sheet_1.pdf]

Supplementary Figures

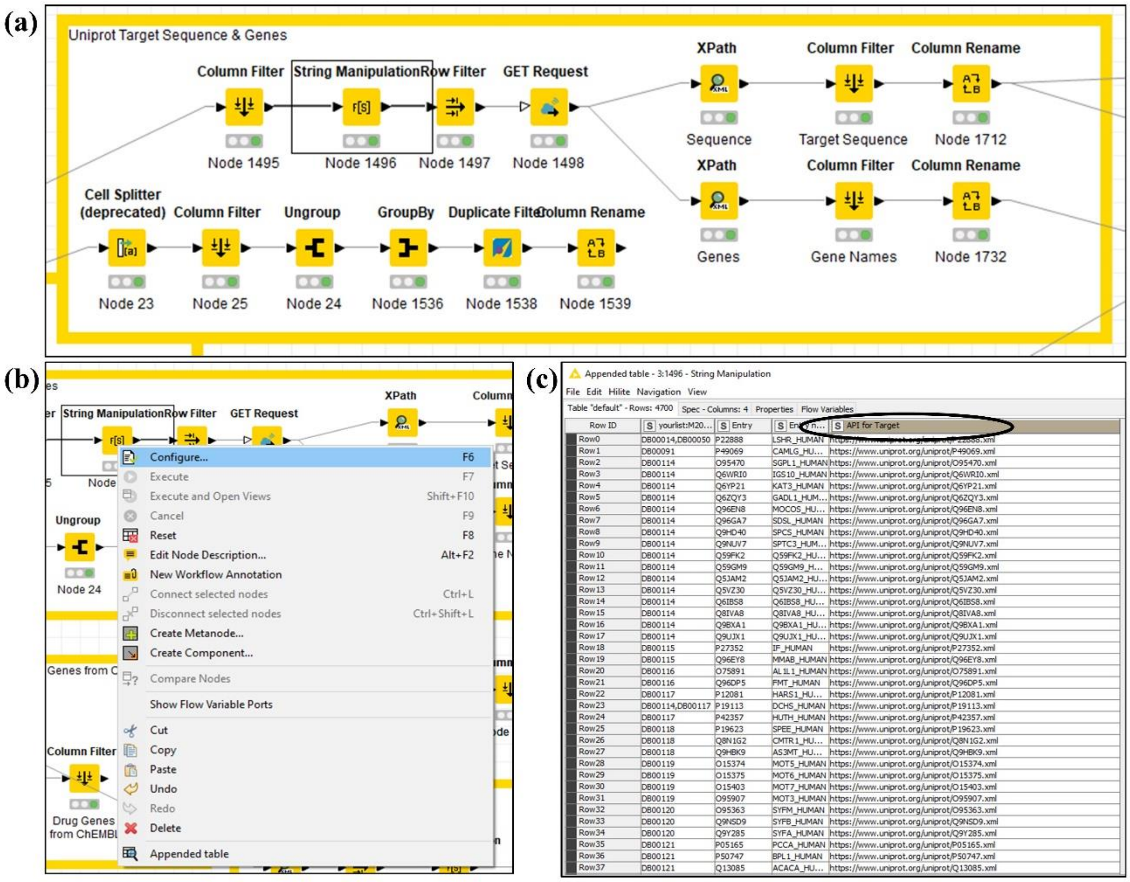

Fig. S1. Shows the process of data accession in KNIME environment a) The protein sequence data is accessed from the Uniprot using the API request made through the String Manipulation node to create the reference links followed by the Get Request node to fetch the data, b) and the setting for the link creation in the String Manipulation node is done in the configuration window, finally c) the created API links for the given targets are shown.

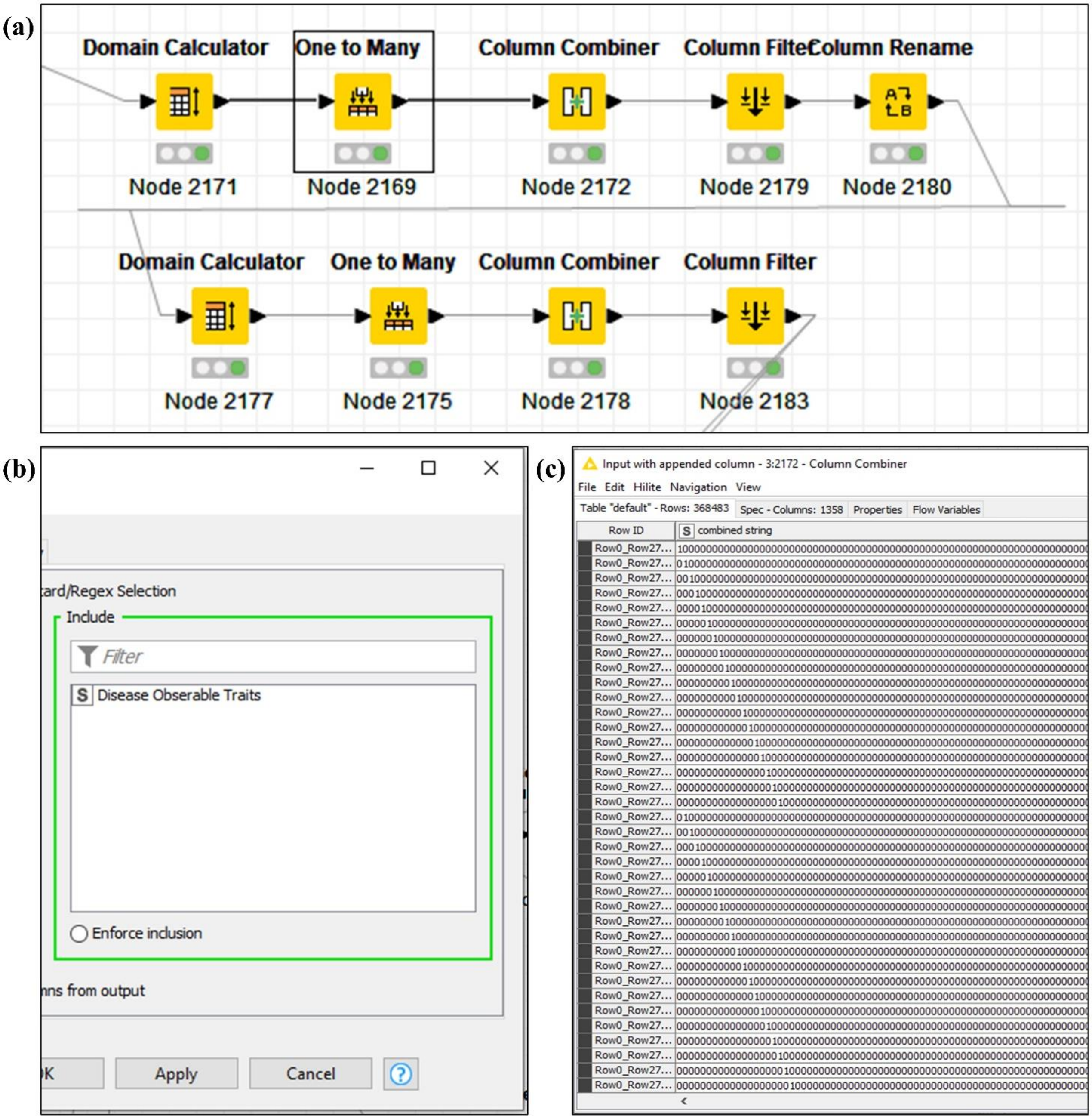

Fig. S2. It shows the one-hot encoding and column combination of the disease observable traits moreover a) to convert the data from textual form to one-hot encoded form One-to-Many node in KNIME Analytics Platform is used. It converts the textual data into string of 0s and 1s followed by the column combiner, b) shows the configuration box of the One-to-Many node where the options for selecting or deselecting the data column, c) and finally the One-hot-encoded combined data in the form of 0s and 1s is shown.

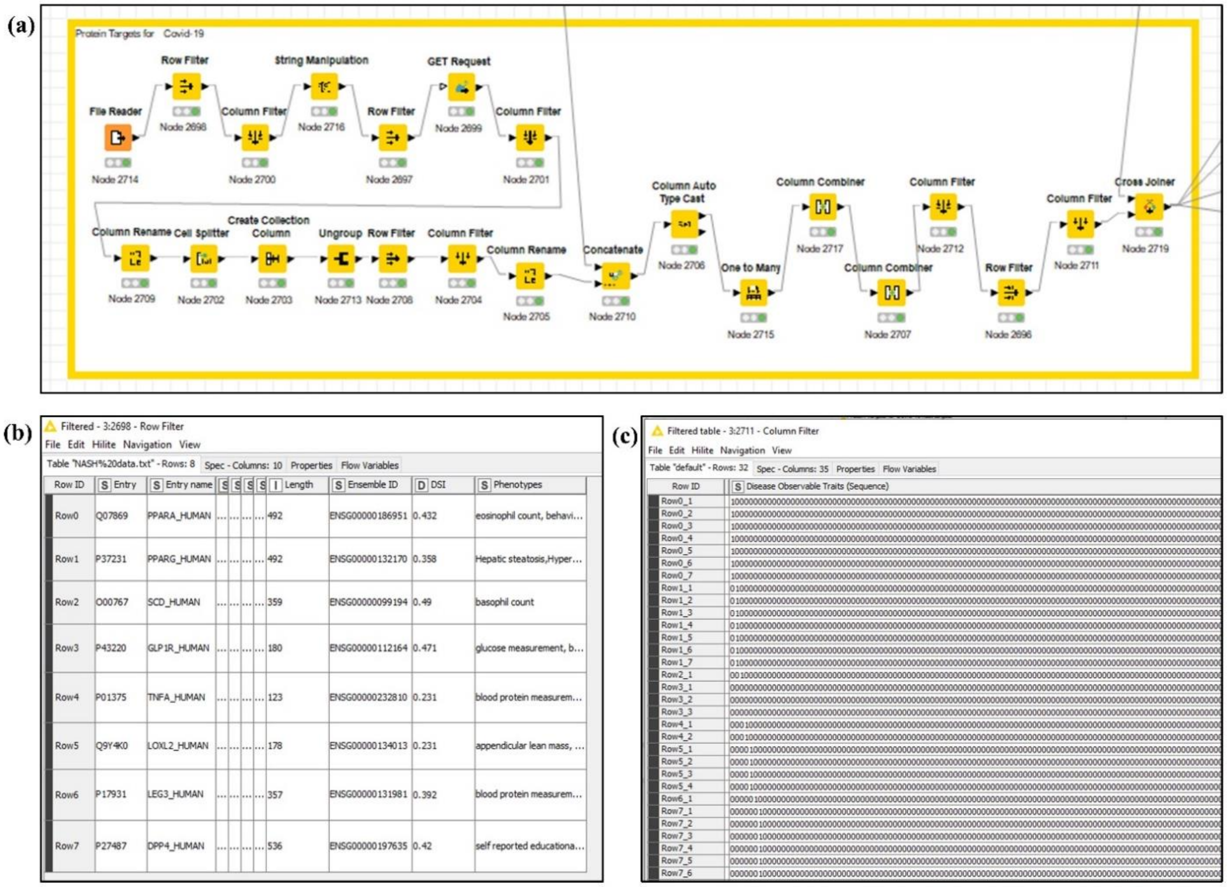

Fig. S3 Process of data preparation for feeding the deployed models for predictions. a) it shows the pipeline, in the form of set of nodes which reads the data through File Reader node and then performs some pre-processing techniques and data retrieval nodes like String Manipulation and Get Request. Finally, after the retrieval and pre-processing the data is merged with the previously prepared drug data with the help of Cross-Joiner, b) the data from the File Reader node is shown which contains the Uniprot IDs, Ensembl IDs, and other disease related feature data, and c) finally the data just before the cross-joiner is shown which looks exactly similar to the data which was used to train the machine learning models.

(a)

(b)

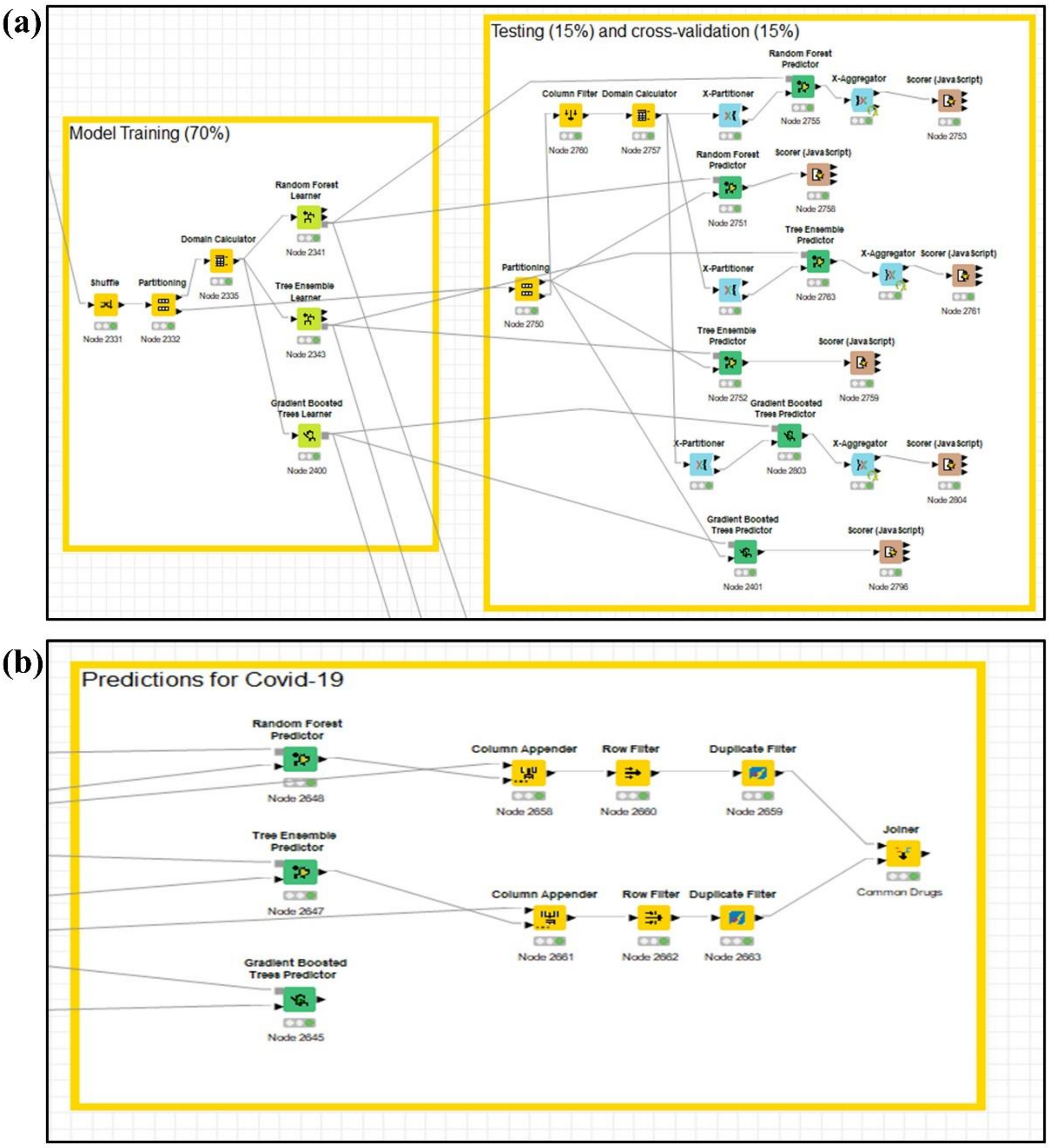

Fig. S4 RF, TE, and GB model training, testing, validation, and predictions setup where a) shows the Model training for the RF, TE, and GB with 70% of the dataset. Target shuffling is performed followed by the data partitioning which is then individually fed to these machine learning models. After training, the models are tested and cross-validated with remaining 30% of the dataset, which is again split into two portions, each of 15%. The testing is performed with 15% along with 10-fold cross validation. b) After the testing and validation is done, the predictions for the Covid-19 are taken by deploying these models. The data is already prepared and the workflow for the data preparation is shown in Fig. S3. The predictions for the confidence range of 0.9 – 1.0 are then further evaluated.
