# Supplementary material for: SperoPredictor: An Integrated Machine Learning and Molecular Docking-Based Drug Repurposing Framework With Use Case of COVID-19
Source: Front Public Health. 2022 Jun 16;10:902123. doi: 10.3389/fpubh.2022.902123 (PMC9244710; doi:10.3389/fpubh.2022.902123)
Supplement: Supplementary file 2 [file Table_1.DOCX]

**Table S1**. Table mentioning the hyper parameters of the classification models used in our study. Here the RF means Random Forest, TE means Tree Ensembl, and GB means Gradient Boosted. Default corresponds to the unchanged value of the Node whereas – corresponds to the value that is not used case.

|  | **Split Criteria** | **Number of Models** | **Tree Depth** | **Node size** | **Learning rate** |
| --- | --- | --- | --- | --- | --- |
| RF | Gini Index | 1000 | 10 | 5 | - |
| TE | Information Gain | 100 | 10 | Default | - |
| GB | Binary Split | 100 | 10 | - | 0.001 |
