# Supplementary material for: SperoPredictor: An Integrated Machine Learning and Molecular Docking-Based Drug Repurposing Framework With Use Case of COVID-19
Source: Front Public Health. 2022 Jun 16;10:902123. doi: 10.3389/fpubh.2022.902123 (PMC9244710; doi:10.3389/fpubh.2022.902123)
Supplement: Supplementary file 3 [file Table_2.DOCX]

**Table S2.** Models training and validation performance. The dataset was split into 70/30 for the training and testing/validation. The training of the models was performed with 70% of the data and resulting performance statistics for testing of the models with 15% of the dataset are mentioned in the table. The remaining 15% of the dataset was used for the 10-fold cross-validation.

| **Algorithm** | **Dataset** | **Accuracy Score** | **MCC** | **F1 Score** | **Precision** | **Sensitivity/Specificity** |
| --- | --- | --- | --- | --- | --- | --- |
| Random Forest | Drug-Target Data | 0.9931 | 0.9863 | 0.9932 | 0.9995 | 0.9870 / 0. 9994 |
|  | 10-fold Cross validation | 0.9924 | 0.9849 | 0.9926 | 0.9997 | 0.9855 / 0.9997 |
| Tree Ensemble | Drug-Target Data | 0.9904 | 0.9809 | 0.9905 | 1.0 | 0.9811 / 1.0 |
|  | 10-fold Cross validation | 0.9894 | 0.979 | 0.9896 | 0.9999 | 0.9796 / 0.9998 |
| Gradient Boosted Learner | Drug-Target Data | 0.505 | 0.0 | 0.6711 | 1.0 | 0.5 / 0.0 |
|  | Cross validation | 0.498 | 0.0 | 0.65 | 0.98 | 0.48/0.0 |
