# Supplementary material for: SperoPredictor: An Integrated Machine Learning and Molecular Docking-Based Drug Repurposing Framework With Use Case of COVID-19
Source: Front Public Health. 2022 Jun 16;10:902123. doi: 10.3389/fpubh.2022.902123 (PMC9244710; doi:10.3389/fpubh.2022.902123)
Supplement: Supplementary file 4 [file Table_3.DOCX]

**Table S3.** Literature based confirmation of predicted drugs and the drugs which were not confirmed from the literature and are proceeded for the molecular docking.

|  | Drug name | DrugBank ID | Prediction Confidence | Targets | Reference. Link |
| --- | --- | --- | --- | --- | --- |
| 1 | Nitrendipine | DB01054 | 0.909 | Q2M2I8 | [85] |
| 2 | Ebselen | DB12610 | 0.916 | \| P09958 \| \| --- \| \| Q2M2I8 \| | [86], [87] |
| 3 | Clidinium | DB00771 | 0.926 | \| P09958 \| \| --- \| \| Q2M2I8 \| |  |
| 4 | Molindone | DB01618 | 0.926 | \| P09958 \| \| --- \| \| Q2M2I8 \| |  |
| 5 | Ezogabine | DB04953 | 0.904 | Q2M2I8 |  |
| 6 | Tecadenoson | \| DB04954 \| \| --- \| \|  \| | 0.912 | Q2M2I8 | [88] |
| 7 | Troxacitabine | DB04961 | 0.94 | Q2M2I8  P09958  O14976 |  |
| 8 | Amonafide | DB05022 | 0.907 | Q2M2I8 |  |
| 9 | Ganaxolone | DB05087 | 0.948 | Q2M2I8  O15393 |  |
| 10 | 16-Bromoepiandrosterone | DB05107 | 0.902 | Q2M2I8 |  |
| 11 | Pracinostat | DB05223 | 0.913 | O14976  O15393  P09958  Q2M2I8 |  |
| 12 | Rolofylline | DB12670 | 0.912 | Q2M2I8 |  |
| 13 | Velusetrag | DB12702 | 0.944 | P09958  Q2M2I8 |  |
| 14 | Balaglitazone | DB12781 | 0.946 | P09958  Q2M2I8  O14976 |  |
| 15 | Gabexate | DB12831 | 0.912 | Q9BYF1  P07711 | [89] |
| 16 | Dihydrexidine | DB12890 | 0.939 | O14976  Q2M2I8  P09958 |  |
| 17 | Dihydralazine | DB12945 | 0.946 | O15393  P07711  Q9BYF1 | [90] |
| 18 | Cortivazol | DB13003 | 0.94 | Q9BYF1  P07711  O15393 |  |
| 19 | Hypericin | DB13014 | 0.901 | Q2M2I8 | [91] |
| 20 | Tiapride | DB13025 | 0.917 | P09958  Q2M2I8 | [92] |
| 21 | Artemisinin | DB13132 | 0.964 | P07711  Q9BYF1 | [93] |
| 22 | Ambroxol acefyllinate | DB13141 | 0.94 | \| O15393  P07711  Q9BYF1 \| \| --- \| | [94] |
| 23 | Potassium gluconate | DB13620 | 0.911 | Q2M2I8 | [95] |
| 24 | Harmaline | DB13875 | 0.943 | \| O14976 \| \| --- \| \| P09958 \| \| Q9BYF1 \| | [96] |
| 25 | Brofaromine | DB13876 | 0.948 | \|  \| \| --- \| \| O15393 \| \| P07711 \| \| Q9BYF1 \| | [97] |
