# Supplementary material for: SperoPredictor: An Integrated Machine Learning and Molecular Docking-Based Drug Repurposing Framework With Use Case of COVID-19
Source: Front Public Health. 2022 Jun 16;10:902123. doi: 10.3389/fpubh.2022.902123 (PMC9244710; doi:10.3389/fpubh.2022.902123)
Supplement: Supplementary file 5 [file Table_4.DOCX]

**Table S4.** Contains the extracted binding sites of the host Covid-19 proteins. In order to extract them, co-crystallized structures were downloaded form Protein Data Bank (RCPDB) and with he help of PyMol visualization tools these sites were extracted and used in our study.

|  | Uniprot ID | PDB ID | Extracted Binding Sites |
| --- | --- | --- | --- |
| 1 | [P07711](https://www.uniprot.org/uniprot/P07711) | 1CS8 | GLY164,SER29,TRP26,HIS163,GLY77,MET75,PHE28 |
| 2 | P09958 | 5MIM | ASP228,ASP191,ASN192,ASP153,GLU236,ASP264,TYR308,SER368 |
| 3 | [Q9BYF1](https://www.uniprot.org/uniprot/Q9BYF1) | 7V8b | ASN53,THR55,ASN58,GLN325,GLN340,MET323,ASN546,ASN322,ASN331,SER420 |
| 4 | Q2M2I8 | 5L4Q | ASN136,GLY132,CYS129,PHE128,ASP127,MET126,ASN181,LYS74,ASP194,PHE195,GLU90,GLY196,MET76,ALA58,PHE57,TYR220,LYS210,VAL78,ASP83,VAL86 |
| 5 | O14976 | 4O38 | ASN136,GLY132,CYS129,PHE128,ASP127,MET126,ASN181,LYS74,ASP194,PHE195,GLU90,GLY196,MET76,ALA58,PHE57,TYR220,LYS210,VAL78,ASP83,VAL86 |
| 6 | O15393 | 7MEQ | GLY439,SER441,SER436,ASP435,CYS437,GLY464 |
