# Supplementary material for: SperoPredictor: An Integrated Machine Learning and Molecular Docking-Based Drug Repurposing Framework With Use Case of COVID-19
Source: Front Public Health. 2022 Jun 16;10:902123. doi: 10.3389/fpubh.2022.902123 (PMC9244710; doi:10.3389/fpubh.2022.902123)
Supplement: Supplementary file 6 [file Table_5.pdf]

Table S5. Showing the way data is collected for pre-processing and then feeding the machine learning models. The Uniprot IDs and Ensembl IDs are collected from the Uniprot along with gene names. Additionally, the DSI values are collected from DisGeNET database and phenotypes are collected from the Monarch database.

	Uniprot ID	Entry Name	Gene names	Ensembl ID	DSI	Phenotypes
1	O15393	TMPS2_HUMAN	TMPRSS2, PRSS10	ENSG00000184012	0.5	3-hydroxypropylmercapturic acid measurement, smoking behavior
2	P07711	CATL1_HUMAN	CTSL CTSL1	ENSG00000135047	0.488	cathepsin L1 measurement
3	P09958	FURIN_HUMAN	FURIN FUR, PACE, PCSK3	ENSG00000140564	0.612	prental longevity, mean arterial pressure, systolic blood pressure, neutrophil count, balding measurement, risk-taking behavior, diastolic blood pressure, healthspan, age at first sexual intercourse measurement
4	Q9BYF1	ACE2_HUMAN	ACE2, UNQ868, PRO1885	ENSG00000130234	0.477	increased cell size, cell separation defects and abnormal colony shape
5	Q2M2I8	AAK1_HUMAN	AAK1, KIAA,1048	ENSG00000115977	0.792	hemoglobin measurement, urate measurement, body height, thyroid stimulating hormone measurement
6	O14976	GAK_HUMAN	GAK	ENSG00000178950	0.53	sepsis, mortality, high density lipoprotein cholesterol measurement, response to vaccine
