# Supplementary material for: SperoPredictor: An Integrated Machine Learning and Molecular Docking-Based Drug Repurposing Framework With Use Case of COVID-19
Source: Front Public Health. 2022 Jun 16;10:902123. doi: 10.3389/fpubh.2022.902123 (PMC9244710; doi:10.3389/fpubh.2022.902123)
Supplement: Supplementary file 7 [file Table_6.DOCX]

Table S6. Free binding energy based prioritized drugs with their docking and redocking scores. Prediction scores from the Machine Learning Models are also given.

|  | Drug Name | DrugBank ID | Covid-19 Targets | Free energy of Binding  (Kcal/mol) | Free energy of Binding  (Kcal/mol) Redocking | Prediction score |
| --- | --- | --- | --- | --- | --- | --- |
| 1 | Cortivazol | DB13003 | Adaptor Protein 2 Associated Kinase 1 (AAK1) | -9.9 | -10.0 | 0.94 |
|  |  |  | Angiotensin-Converting Enzyme 2 (ACE2) | -10 | -10.0 |  |
|  |  |  | Furin | -9.4 | -9.4 |  |
| 2 | Velusetrag | DB12702 | Adaptor Protein 2 Associated Kinase 1 (AAK1) | -9.1 | -8.6 | 0.944 |
|  |  |  | Cyclin-G-Associated Kinase (GAK) | -10 | -10 |  |
| 3 | 16-alpha Bromoepiandrosterone | DB05107 | Cyclin-G-Associated Kinase (GAK) | -9.0 | -9.0 | 0.902 |
|  |  |  | Furin | -8.7 | -8.7 |  |
| 4 | Balaglitazone | DB12781 | Adaptor Protein 2 Associated Kinase 1 (AAK1) | -9.9 | -10.1 | 0.946 |
|  |  |  | Cyclin-G-Associated Kinase (GAK) | -9.6 | -9.7 |  |
|  |  |  | Furin | -9.1 | -9.1 |  |
| 5 | Ganaxolone | DB05087 | Adaptor Protein 2 Associated Kinase 1 (AAK1) | -9.1 | -8.9 | 0.948 |
| 6 | Rolofylline | DB12670 | Cyclin-G-Associated Kinase (GAK) | -9.1 | -9.0 | 0.912 |
